# Are Mesenchymal Cells Indeed Pluripotent Stem Cells or Just Stromal Cells? OCT-4 and VSELs Biology Has Led to Better Understanding

**DOI:** 10.1155/2013/547501

**Published:** 2013-09-25

**Authors:** Deepa Bhartiya

**Affiliations:** Stem Cell Biology Department, National Institute for Research in Reproductive Health, Parel, Mumbai 400 012, India

## Abstract

Stem cells have excited researchers because of their potential to regenerate. However, which stem cells will be the best candidate for regenerative medicine remains an enigma. Compared to pluripotent stem cells with associated risks of immune rejection and teratoma formation, adult stem cells especially the mesenchymal stem cells (MSCs) are hyped to be a suitable alternate since they also exhibit pluripotent properties. This review shows that there is a subpopulation of pluripotent very small embryonic-like stem cells (VSELs) among MSCs culture. The two populations differ from each other in expression pattern of OCT-4. VSELs exhibit nuclear OCT-4A, whereas the MSCs have cytoplasmic OCT-4B, similar to our earlier findings in testis and ovary. Pluripotent VSELs with nuclear OCT-4A exist in various adult body organs, and the immediate progenitors express cytoplasmic OCT-4B which is eventually lost as the cell differentiates further. To conclude it is essential to discriminate between nuclear and cytoplasmic OCT-4 expression and also to acknowledge the presence of VSELs.

## 1. Introduction

Stem cells represent a novel cell type in the body which has the potential to regenerate any worn out tissue and maintain tissue homeostasis. Stem cells can be multiplied in large numbers *in vitro *and may serve to replace the damaged cells for regeneration rather than the existing means of managing diseases by treating the damaged cells with drugs. Stem cells are broadly classified based on their source into embryonic (hESCs) and adult (ASCs) stem cells. Embryonic stem cells are pluripotent in nature and can be differentiated into 200 odd cell types in the body belonging to the three germ layers, namely, ectoderm, endoderm, and mesoderm. On the other hand adult stem cells are isolated from adult body tissues and are multi- to unipotent in nature. Since the initial isolation of hES cell lines [1], there has been a divide amongst the embryonic and adult stem cell biologists. It has been the endeavor of the adult stem cell biologists to demonstrate that ASCs are equally good compared to hES cells, and thus hES cell research is not required (because of associated ethics since spare human embryos are used and manipulated). In January 2013, hES cell biologists were greatly relieved, when US Supreme Court refused to hear a case that could have prohibited government funding for hES cells [2]. Various approaches have been used to demonstrate that ASCs can replace hES cells. In particular with the ability to reprogram adult somatic cells to pluripotent state by iPS technology, the lobby against hES cells has become still more strong. Another issue that has been highlighted is that mesenchymal stem cells are pluripotent and besides the differentiation into mesoderm can also transdifferentiate into ectoderm and endoderm [3] and is the focus of this special issue.

Mesenchymal stem cells (MSCs) are spindle-shaped-plastic-adherent cells that can be isolated from the fetus, extra embryonic tissues; and adult organs including bone marrow and several other body tissues. MSCs were first described by Friedenstein and group [4] as hematopoietic supportive mesenchymal stromal cells of bone marrow. Owen and Friedenstein [5] proposed that these cells may be termed mesenchymal stem cells as they had the ability to differentiate into lineages of mesenchymal tissues including bone, cartilage, tendon, ligament, marrow stroma, adipocytes, dermis, muscle, and connective tissue. However, whether they are a true, stem cell still remains controversial. The names mesenchymal stem or stromal cells are interchangeably used in the literature. The International Society for Cellular Therapy (ISCT) has recommended that these spindle-shaped, plastic-adherent cells be termed, mesenchymal stromal cells [6]. It has been proposed that a yet unidentified stem cell may exist amongst the MSCs, but MSCs themselves must be termed mesenchymal stromal cells [7]. The recent literature suggests that MSCs are a crucial component of the niche for the HSCs in the bone marrow [8, 9].

 MSCs undergo lineage-specific differentiation into mesoderm, but the ability to transdifferentiate into other lineages remains controversial. Various groups have published that MSCs can transdifferentiate into ectodermal and mesodermal lineages including hair [10], pancreatic islets [11, 12], hepatocytes [13], and neurons [14, 15]. Greco et al. [16] have further shown that a similar regulatory mechanism for OCT-4 exists among ES cells and MSCs. However, this remains highly controversial especially because the functional properties of MSCs transdifferentiated into ectoderm and endoderm are not as expected. Similarly Osonoi et al. [17] reported that human dermal fibroblasts are able to differentiate directly to all 3 germ layer derivatives that is, neurons (ectodermal), skeletal myocytes (mesodermal), and insulin-producing cells (endodermal). They exhibit nestin, desmin, and insulin when exposed to specific cocktail of growth factors. Thus it is felt that achieving transdifferentiation on the basis of immunolocalization or presence of transcripts may not suffice. Rather, evidence needs to be generated regarding the functional maturation—which has not yet been achieved.

There are two main facets of stem cells biology that have indeed baffled researchers and have led to this confusion about the functional attributes of MSCs. These include (i) OCT-4 biology and (ii) presence of a subpopulation of pluripotent very small ES-like stem cells (VSELs) amongst MSCs.

## 2. Oct-4 Biology and Pluripotency

Oct-4 is the most crucial POU domain transcription factor responsible for maintaining the self-renewal and pluripotent properties of stem cells including inner cell mass, embryonic stem cells, embryonic germ cells, and embryonic carcinoma cells. Oct-4, Nanog, Sox2, and FoxD3 together form an interconnected autoregulatory network to maintain ES cells pluripotency and self-renewal [18]. Oct-4-deficient mice do not develop beyond blastocyst stage due to lack of pluripotent inner cell mass cells [19]. Oct-4 is downregulated with loss of pluripotency, and knockdown of Oct-4 in ES cells results in differentiation [20, 21]. It has two major isoforms Oct-4A and Oct-4B of which only Oct-4A is responsible for the pluripotent state, whereas no biological function has been associated with Oct-4B isoform [22]. Atlasi et al. [23] reported another Oct-4 spliced variant which is primarily expressed in the pluripotent stem cells and is downregulated following differentiation; however, its function is still not clear [24]. It becomes crucial to discriminate between the various isoforms while concluding pluripotent state of a cell [23, 25]. But stem cell biologists have overlooked this aspect during their studies, have reported Oct-4 in several nonpluripotent cell types, and have resulted in a great deal of confusion [24, 26]. Similarly, there was a lot of excitement recently when various groups reported derivation of pluripotent ES-like cultures from adult testicular biopsies in mice [27–30] as well as in men [31–33] by spontaneous reprogramming of adult spermatogonial stem cells without any genetic modification. However, Warthemann et al. [34] have shown that false-positive antibody signals for OCT-4A in testis-derived cells may have led to erroneous data and misinterpretations.

Oct-4 has been reported in several somatic cell types, placenta, amniotic and cords-derived cells, and also in primary tumor tissues (refer to Supplemental Table 1 in [35]). Zangrossi et al. [36] demonstrated the presence of Oct-4 in peripheral blood and thus challenged whether OCT-4 should really be a marker for pluripotency. Greco et al. [16] showed that OCT-4 functions through similar pathway in human MSCs and ES cells. However, all these reports studied Oct-4 and failed to discriminate between the alternatively spliced Oct-4 transcripts.

In an attempt to clarify the confusion between ASCs and ESCs with respect to Oct-4 expression, Lengner et al. [35] deleted Oct-4 in several tissues with rapid turnover including intestine, bone marrow, hair follicle, liver, or CNS but found no effect on tissue maintenance or injury-induced regeneration. Thus they concluded that Oct-4 expressing cells are not required for maintaining homeostasis in adult body organs. They further discussed that somatic OCT-4 expression could be due to nonspecific staining since the amount of mRNA was very low in somatic cells compared to the ES cells and invariably amplified after 30–40 cycles of PCR amplification.

However, their concluding statement is rather intriguing. They do not deny presence of Oct-4 in adult body tissues, but the levels are very low compared to the ES cells. This is very true for the pluripotent very small ES-like stem cells (VSELS) in adult body tissues.

## 3. Pluripotent Stem Cells in Adult Body Tissues

Very small embryonic-like stem cells (VSELs) represent a very promising group of stem cells which have the potential to bring together embryonic and adult stem cell biologists. These are pluripotent stem cells in adult body tissues. They exhibit pluripotent characteristics including nuclear Oct-4 albeit at very low level compared to hES cells. However, they can be isolated from autologous source and do not form teratoma in mice (thus all the three major issues associated with hES cells including using spare human embryos to derive hES cell lines, immune rejection, and risk of teratoma formation are taken care of). They are easily mobilized in response to any injury, maintain life-long homeostasis [37, 38], and are also considered as embryonic remnants responsible for various cancers in the body [39], as proposed 150 years ago by Rudolf Virchow and Julius Conheim. Pioneering work done by Professor Ratajczak and his group have shown that pluripotent, VSELs exist in various adult body tissues [40] and are possibly the primordial germ cells or their precursors which rather than migrating only to the gonadal ridges during early embryonic development migrate to various body organs and persist throughout life.

**Figure 1:**
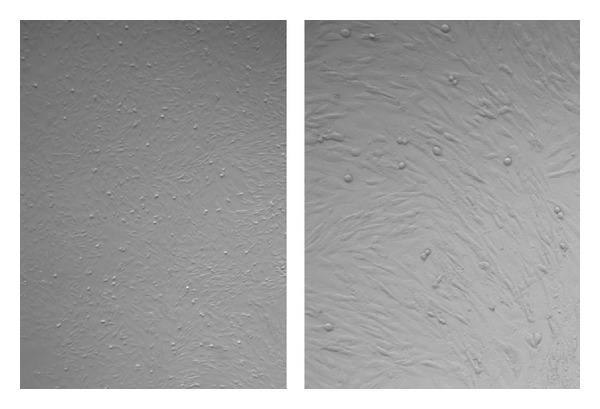
Bovine bone marrow culture to propagate mesenchymal cells. Note that the culture comprises a subpopulation of spherical cells along with the MSCs. These small round cells are possibly the VSELs.

The confusion in the literature about presence of Oct-4 in adult body tissues is actually because of VSELs. VSELs with nuclear OCT-4 exist in various tissues and give rise to the tissue-specific progenitors which further differentiate into tissue-specific cell types. As the VSELs start differentiating, OCT-4 is observed in the cytoplasm and as the cells differentiate further, it is eventually lost. Our work on mammalian gonads has shown that indeed VSELs with nuclear OCT-4 and their immediate progenitors spermatogonial stem cells (SSCs) in testis [40] and ovarian germ stem cells (OGSCs) in the ovary have cytoplasmic OCT-4 [41]. We used a polyclonal antibody against OCT-4 which detects expression for both the isoforms (i.e. nuclear and cytoplasmic) and has shown that VSELs have nuclear Oct-4, and once differentiation is initiated in the progenitors, OCT-4 is cytoplasmic. Q-PCR analysis clearly shows the abundance of Oct-4B over Oct-4A. In order to show presence of pluripotent VSELs in the adult mammalian gonads, we have always shown the presence of Oct-4A rather than Oct-4. We also reported the presence of VSELs in the discarded pellet of RBCs during volume reduction step while processing cord blood and bone marrow [42] and also in MSCs culture (Figure 1).

**Figure 2:**
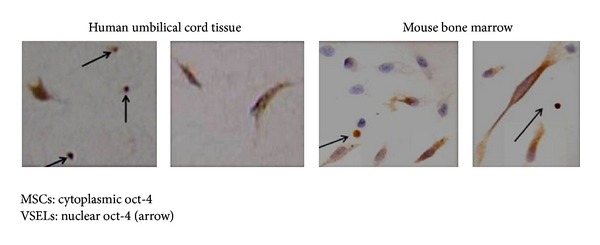
Immunolocalization of OCT-4 in MSCs and VSELs in human umbilical cord tissue sections and mouse bone marrow smears. Note that the round spherical VSELs have nuclear OCT-4, whereas the MSCs have cytoplasmic OCT-4.

Umbilical cord tissue, especially Wharton's jelly and bone marrow, is considered as a rich source of MSCs. Immunohistochemical studies of Wharton's jelly clearly show the presence of a subpopulation of VSELs amongst the MSCs ( Figure 2 [42]). Similarly, early passages of MSCs from mouse bone marrow show the presence of VSELs as a distinct subpopulation (personal observations). Interestingly OCT-4 showed nuclear expression in Wharton's jelly VSELs and was cytoplasmic in the MSCs. Similarly, Taichman et al. [43] demonstrated that VSELs could be on top of hierarchy for mesenchymal stem cells (MSCs) in mice. We made a case for VSELs present in the mammalian testis [44] that may actually give rise to the ES-like colonies during testicular tissue cultures [27–33]. Observations made by Lengner et al. [35] are indeed true because Oct-4 is expressed at very low levels in the VSELs (detected only after >35 cycles during RT-PCR) compared to ES cells (detected after 20–25 cycles during RT-PCR), and the immediate progenitors that is, the adult stem cells that exist in various adult tissues, express cytoplasmic Oct-4 which is eventually lost as cells become more committed. Berg and Goodell [45] coauthored a preview on the Lengner study and correctly summarized in the first sentence that “absence of evidence is not evidence of absence” or stated another way “one cannot prove a negative.” They also hinted to the existence of a stem cell population that was not tested in the studies reported and now we understand that it was possibly the VSELs.

Nayernia et al. [46] first reported that BM stem cells/MSCs can transdifferentiate into male germ cells both *in vitro *and *in vivo*. They transplanted BM cells into busulphan treated mice and observed colonization and proliferation but no differentiation beyond premeiotic spermatocytes stage. After this several groups have reported restoration of testicular function by transplanting MSCs. Lue et al. [47] transplanted GFP-tagged BM cells into the testicular interstitium and tubules of wild type mice and reported that the transplanted cells differentiate into Leydig cells, Sertoli cells, and also into germ cells. Similarly, Aziz et al. [48] also reported that bone marrow-derived MSCs when transplanted into the rete testis of busulphan-treated azoospermic rats transdifferentiate into spermatids and spermatocytes. Sabbaghi et al. [49] studied the ability of BM derived MSCs in revival of sperm in rat model for testicular torsion. They have reported that transplantation of MSCs via rete testis can revive spermatogenesis. Cakici et al. [50] also recently reported that fertility is restored in azoospermic rats by injecting adipose-derived MSCs. But this whole body of the literature is confusing because these studies fail to acknowledge the presence of VSELs in mammalian testis which are indeed resistant to busulphan treatment. VSELs are also resistant to damage induced by radiation because of their quiescent nature [51]. VSELs persist in busulphan treated testis and possibly differentiate into germ cells/sperm in the presence of growth factors/cytokines secreted by the transplanted MSCs [52].

To conclude, we propose that MSCs indeed arise from VSELs [53] in agreement with earlier reports by Taichman et al. [43] and are multipotent implying that they can give rise to various mesodermal cell types. Their pluripotent properties implying transdifferentiation are questionable and whatever minimal transdifferentiation that is reported may actually be due to the existing subpopulation of VSELs. The very presence of MSCs in so many diverse body tissues forces us to think that they actually represent a highly specialized ground substance or the microenvironment (source of growth factors and cytokines) for the VSELs and their progenitors to maintain life-long tissue homeostasis and are capable of immune modulation. The growth factors and cytokines secreted by the MSCs keep the VSELs under quiescent state and maintain normal proliferation and differentiation. But with increased age, MSCs function is compromised resulting in uncontrolled proliferation of stem cells at any level resulting in increased incidence of cancers. If VSELs function is disrupted the tumors are more embryonic in nature and more lethal. Nature of the tumors will vary if more committed progenitors function gets disrupted due to the altered secretome of the niche providing cells. Thus the interaction of MSCs with VSELs and the tissue-committed stem cells “progenitors” and age related changes in the MSCs secretome warrants further investigations.
